# Effects of bathing in different hot spring types on Japanese gut microbiota

**DOI:** 10.1038/s41598-024-52895-7

**Published:** 2024-01-28

**Authors:** Midori Takeda, Jungmi Choi, Toyoki Maeda, Shunsuke Managi

**Affiliations:** 1 Urban Institute & Department of Civil Engineering, Kyushu University, 744 Motooka, Nishi-ku, Fukuoka, 819-0395 Japan; 2 Department of Internal Medicine, Kyushu University Beppu Hospital, 4546 Tsurumihara, Beppu, Oita, 874-0838 Japan

**Keywords:** Metagenomics, Microbiome, Public health

## Abstract

Hot springs have been used for a variety of purposes, including the treatment and amelioration of illness and recreation. Japan has ten different types of therapeutic springs (described here as spa types), which are traditionally believed to have different efficacy. However, more research must be conducted to determine how they affect healthy people. Therefore, this study focused on the gut microbiota and aimed to investigate changes in the gut microbiota in healthy people after bathing in different spa types. Using Beppu's hot springs (simple, chloride, bicarbonate, sulfur, and sulfate types), 136 healthy Japanese adults living in the Kyushu area participated in the study and bathed in the same hot spring for seven days. Fecal samples were collected before and after the 7-day bathing period, and the relative abundance of the gut microbiota was determined by 16S rRNA sequencing. The results showed that the relative abundance of *Bifidobacterium bifidum* increased significantly after seven consecutive days of bathing in the bicarbonate spring. Significant increases in other gut microbiota were also observed after bathing in simple, bicarbonate, and sulfur springs. These results suggest that bathing in different hot springs may affect the gut microbiota in healthy individuals differently.

## Introduction

The Beppu hot spring area in Kyushu has over 2000 hot springs, the most in Japan regarding sources and outflow, featuring a diverse range of thermal waters from these volcanic systems. Historically, these natural springs, collectively known as Beppu Hattou, have played a significant role in local development and balneotherapy practices^1–5^.

Hot springs have long been used for empirical medical purposes in many parts of the world. Known as balneotherapy, bathing or drinking mineral or hot spring water has been used as an adjunct to treatment or for pain relief^1,2^. Some previous studies have reported that hot spring therapy may benefit sleep quality and quality of life in patients with musculoskeletal and skin diseases^3–5^. Several papers have also reported the effectiveness of bathing in hot springs in alleviating cardiovascular disease and hypertension^6–12^. Hot spring use is recommended for medical patients who have particular symptoms, including symptoms of the gastrointestinal tract, respiratory system, ear, nose, throat, or skin; gynecological symptoms; and rheumatological symptoms^13–15^. Recent advances in Balneotherapy research have highlighted its multifaceted role in managing various health conditions, particularly rheumatological and dermatological diseases. For instance, studies by Forestier et al.^16,17^ and Fioravanti et al.^18,19^ have elucidated the therapeutic benefits of Balneotherapy in conditions like osteoarthritis and fibromyalgia, indicating improvements in pain management and functional capacity. Additionally, emerging research has begun to unravel the complex interactions between Balneotherapy and the human microbiome. Nicoletti et al.^20^ and Barnich et al.^21^ have provided insights into how mineral water and its constituents can influence the gut and skin microbiota, leading to potential health benefits. Moreover, recent findings by Manara et al.^22^ demonstrate the modulation of the skin and gut microbiome in psoriatic patients through thermal therapy. Thirion et al.^23^ showed that balneotherapy impacted gut microbiota in atopic dermatitis, linking to disease severity and improvement. These studies underscore the importance of mineral water's microbiological components and their systemic effects, paving the way for novel therapeutic approaches in Balneotherapy.

In Japan, hot springs are defined by the Hot Spring Law^24^ enacted by the government in 1948 and the Standard Methods of Analysis for Mineral Springs by the Ministry of the Environment^25^. According to the laws, hot springs are defined as 'hot water, mineral water, steam, and other gases (excluding natural gas composed mainly of hydrocarbons) that emanate from the ground, which is above 25 °C or has a certain level of a specific substance^24–26^. In addition, the Ministry of the Environment Japan classified therapeutic hot springs, which are considered to have medicinal properties, into ten major categories defined by the amount and concentration of the contained substances^25,27^. These categories are called spa types or ‘*sen-shitsu*’ in Japanese. The spa types and their criteria are as follows: simple, > 25 °C, dissolved substance < 1 g/kg; chloride, dissolved substance (Cl^-^)≧ 1 g/kg; carbonate, dissolved substance (HCO_3_^-^) ≧ 1 g/kg; sulfate, dissolved substance (SO_4_^2-^) ≧ 1 g/kg; carbon dioxide, CO_2_ ≧ 1000 mg/kg; iron, Fe^2+^  + Fe^3+^ ≧ 20 mg/kg; acidic, H^+^ ≧ 1 mg/kg; iodine, I^-^ ≧ 10 mg/kg; sulfur, S ≧ 2 mg/kg; and radioactive, Rn ≧ 30 × 10^–10^ Ci = 111 Bq/kg^25,27,28^. Each therapeutic spring has traditionally been said to have its specific benefits^26^, there are few studies that scientifically verify these effects or the differences in effects between different spring types.

Therefore, this study aimed to investigate the effects of bathing in different hot springs on the gut microbiota in healthy individuals. It may provide new insights into the effects of bathing in different therapeutic hot springs.

## Methods

### Ethics approval and consent to participate

This study was approved by the Ethical Committees of Urban Institute (permission number: 230609-01). All subjects were informed of the purpose of this study, and written consent was obtained from all subjects. All studies were performed in accordance with the Declaration of Helsinki.

### Study subjects and bathing procedures

This study was conducted between June 2021 and July 2022 and included 136 participants (80 males and 56 females). Inclusion criteria were men and women who were at least 18 years old and under 65 years old, gave their free written consent, had not been diagnosed with a chronic disease, and had not bathed in a hot spring within 14 days prior to allocation. In addition, the exclusion criteria excluded individuals who did not complete the designated number of bathing days and those with highly irregular eating habits. Healthy Japanese adults between the ages of 20 and 65 living in the Kyushu area were recruited and asked to bathe in the same hot spring for seven consecutive days. Each participant chose a hot spring facility and was required to soak in the same bathtub at the same hot spring facility. The total time spent in the bathtub was at least 20 min daily, with free breaks. Regarding diet and lifestyle, the participants were asked to lead an everyday life and refrain from overeating and excessive drinking.

### Fecal sample collection and processing

Pre- and postexperimental fecal samples were collected by the participants themselves using a fecal collection brush type kit (TechnoSuruga Laboratory Co., Ltd., Japan). The fecal samples were sent to Symbiosis Solutions Inc. (Tokyo, Japan) without any temperature control after being suspended in a guanidine thiocyanate solution [100 mM Tris–HCL (pH 8.0), 40 mM Tris–EDTA (pH 9.0), 4 M guanidine thiocyanate, 0.001% bromothymol blue]. Symbiosis Solutions Inc. performed the DNA extraction, sequencing, 16S rRNA data analysis, and occupancy calculations for each bacteria according to previously reported methods^29^. The gut microbiota were identified at the genus level and analyzed to determine the 20 most common genera in all participants.

### Statistical analysis

Statistical analyses were performed using R software (version 4.3.1) and Prism 9. The Wilcoxon matched-pairs signed rank test was used to determine the change in the relative abundance of each intestinal bacterium before and after hot spring bathing. No normality test was performed due to the nonparametric nature of the method. For differential abundance analysis, the false-discovery rate (FDR) method was used to correct p values for multiple testing, and q values were calculated using the Benjamini‒Hochberg method. Statistical significance was set at p < 0.05 and q < 0.05. Statistical analysis was performed on groups of n≧10 (simple, chloride, bicarbonate and sulfur).

## Results

### Data summary

**Figure 1:**
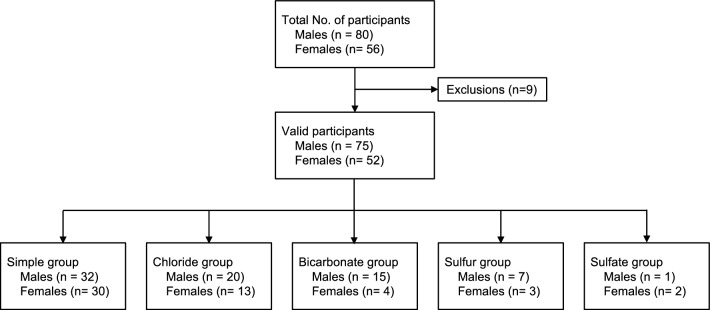
Flowchart of the study population. Of the 136 participants, nine were excluded due to insufficient number of bathing days or fecal samples. The final analysis included 127 individuals, and the numbers of participants are listed for each spa type. Sulfate springs were excluded from the analysis due to the small number of participants.

Of the 136 participants, we excluded 9 participants (ID 7, 19, 25, 35, 121, 124, 136, 185, 190) who had an insufficient number of bathing days or who could not be measured due to a lack of fecal specimens (Fig. 1). Excluding these 9 participants, 127 valid participants (75 males and 52 females) were analyzed to evaluate the effect of the spa type on the gut microbiota (Fig. 1). Basic statistics for each group are shown in Table 1. The ordinary one-way analysis of variance (ANOVA) results for the age of each group were not significantly different, and the p value was 0.324. Table 2 lists the hot spring facilities, spa types, and numbers of valid participants.

**Table 1:** Characteristics of the study population.

	Simple	Chloride	Bicarbonate	Sulfur	Sulfate	P value
Age (years)^1^	39.3 (11.2)	43.9 (11.3)	40.5 (12.2)	41.6 (11.6)	40.0 (15.1)	0.324
N^1^	62 (49%)	33 (26%)	19 (15%)	10 (8%)	3 (2%)	

^1^Mean (SD); n/N (%).

**Table 2:** Summary of the hot spring facilities, types of hot springs, and number of valid participants in this study.

Names of hot spring facilities	Spa type	Total	Male	Female
Kamenoi Hotel Beppu	Simple	34	18	16
Ryouchiku-Bettei Spa & Resort	Simple	13	8	5
Yuya Ebisu	Simple	7	1	6
Hotel Sun Valley ANNEX	Simple	4	3	1
ANA Intercontinental Beppu Resort & Spa	Simple	4	2	2
Amane Resort Seikai	Chloride	7	6	1
Onsenkaku	Chloride	1	1	0
Hotel New Tsuruta	Chloride	6	6	0
Kurodaya	Chloride	10	3	7
Beppu Showaen	Chloride	7	4	3
Beppu Asahiya	Chloride	2	0	2
Hotel Sea Wave Beppu	Bicarbonate	8	4	4
Hotel Shiragiku	Bicarbonate	4	4	0
Beppu Kouraku	Bicarbonate	3	3	0
Bokai Hotel	Bicarbonate	4	4	0
Okamotoya Ryokan	Sulfur	5	3	2
Myoban Yunosato	Sulfur	5	4	1
Yumotoya Ryokan	Sulfate	3	1	2
Total		127	75	52

### Changes in the relative abundance of gut microbiota before and after hot spring bathing in each spa type

**Figure 2:**
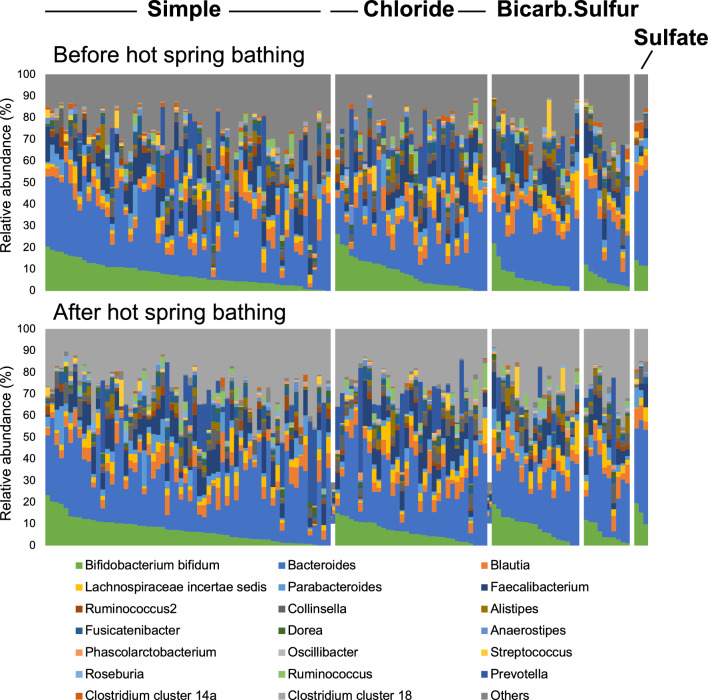
Comparisons of the relative abundance of gut microbiota before and after hot spring bathing. One of the stacked bars corresponds to one individual. Each hot spring bathing group is shown as a single mass, arranged starting with the individual with the highest *Bifidobacterium bifidum* value on the left. The upper row indicates before bathing, and the lower row indicates after bathing. The order of individuals is not identical in the upper and lower rows. ‘Bicarb.’ indicates ‘Bicarbonate’.

The relative abundance of the gut microbiota of 127 subjects before and after bathing in a hot spring is shown in the stacked bar graph in Fig. 2. The upper row is before bathing, and the lower row is after bathing for each group. The individuals are listed from left to right in order of the relative amount of *Bifidobacterium bifidum*, so the order of individuals in the upper and lower rows is different. The relative abundance of each gut microbiota and the spa type for the 127 valid participants are listed in Supplementary Table S1.

**Figure 3:**
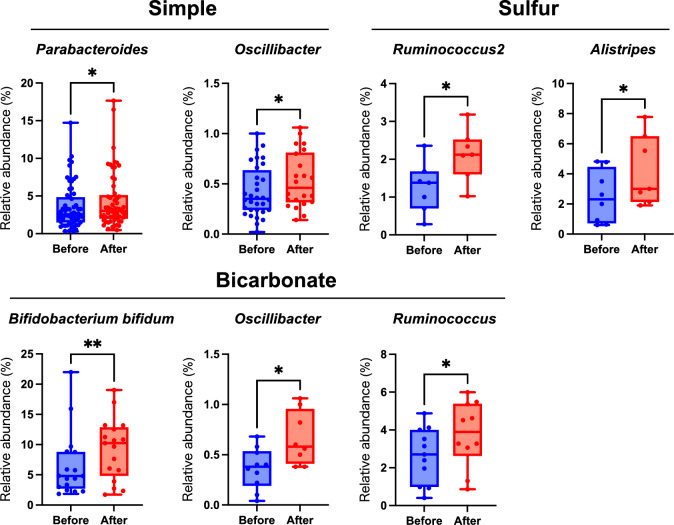
Bacteria significantly different before and after bathing in hot springs of each spa type. In the box plots, the box boundary closest to zero indicates the 25th percentile, the line within the box marks the mean, and the box boundary farthest from zero indicates the 75th percentile. Whiskers above and below the box indicate the max and min. Dots indicate the value of each individual. The spa type and the name of the bacteria are listed above each plot. The Wilcoxon matched-pairs signed rank test was used to analyze comparisons before and after spa bathing (*p < 0.05, **p < 0.01).

Next, before-and-after comparisons for 20 genera of bacteria in each of the four groups (simple, chloride, bicarbonate, and sulfur) were made using the Wilcoxon matched-pairs signed rank test. The respective p values are listed in Supplementary Table S2. An FDR correction was performed to account for multiple tests, and the corrected p values were calculated as q values. The Benjamini‒Hochberg method was used for this calculation. Bacteria and groups with significantly altered relative abundance are listed in Table 3, along with p values and q values. The results showed that seven bacteria were significant, and all were increased after bathing. *Parabacteroides* and *Oscillibacter* increased in the simple group; *Bifidobacterium bifidum*, *Oscillibacter*, and *Ruminococcus* increased in the bicarbonate group; and *Ruminococcus2* and *Alistipes* increased in the sulfur group (Table 3 and Fig. 3). In summary, *Oscillibacter* was found to be a common bacterium of simple and bicarbonate bathing, while the other five increased bacteria were unique to one type.

**Table 3:** List of bacteria that showed significant changes for each hot spring type before and after bathing.

Spa type	Bacteria	Mean	p value	q value	Sig	n
Simple	*Parabacteroides*	+ 0.701	0.0379	0.0410	Yes	57
Simple	*Oscillibacter*	+ 0.141	0.0314	0.0410	Yes	20
Bicarbonate	*Bifidobacterium bifidum*	+ 2.796	0.0079	0.0365	Yes	17
Bicarbonate	*Oscillibacter*	+ 0.305	0.0156	0.0365	Yes	8
Bicarbonate	*Ruminococcus*	+ 1.286	0.0410	0.0410	Yes	10
Sulfur	*Ruminococcus 2*	+ 0.866	0.0313	0.0410	Yes	7
Sulfur	*Alistipes*	+ 1.486	0.0156	0.0365	Yes	7

The mean column indicates the increase in relative amount after bathing in the hot spring.

*Bifidobacterium bifidum* exhibited the largest change, with an increase of 2.796% after bathing in bicarbonate hot springs (Table 3). *Parabacteroides* was increased by 0.701% after bathing in simple springs. *Oscillibacter* was increased by 0.141% in simple springs and 0.305% in bicarbonate springs, and *Alistipes* was increased by 1.486% in sulfur springs. *Ruminococcus* was increased by 1.286% in bicarbonate springs. *Ruminococcus2* was increased by 0.866% in sulfur springs.

The study revealed that different hot spring types resulted in modifications in gut microbial composition after bathing, significantly increasing specific bacteria. These findings highlight the potential role of unique mineral properties in forming microbiome responses and suggest a targeted impact on microbes resulting from the unique chemical profile of each hot spring type.

## Discussion

In this study, for the first time, we examined the effects of bathing in hot springs of different spa types on the gut microbiota of Japanese people. The largest change in the relative abundance was observed in *Bifidobacterium bifidum*, which increased significantly after bathing in bicarbonate hot springs.

In addition to *Bifidobacterium bifidum*, significant increases in *Oscillibacter* and *Ruminococcus* were also observed in the bicarbonate group, and there were significant increases in *Parabacteroides* and *Oscillibacter* in the simple group and in *Ruminococcus2* and *Alistipes* in the sulfur group. No significant bacteria were identified in the chloride group.

*Bifidobacterium bifidum* is a beneficial bacterium that has been reported to improve constipation and glucose tolerance and provide protection from enteropathogenic infection^30–32^. It suggests the possibility of improving constipation, glucose tolerance, and immune function. However, detailed studies using each health indicator as an outcome are needed to demonstrate these health-improving effects of hot spring bathing. For the other increased bacteria, there are reports of both positive and negative effects on health. *Parabacteroides* is also believed to have a positive effect because it is enriched in centenarians^33^, while worsening of amyotrophic lateral sclerosis (ALS) symptoms in mice with *Parabacteroides distasonis* or *Ruminococcus torques* has been reported^34^. *Oscillibacter* and *Alistipes* were reported to be causally related to lower triglyceride concentrations^35^. Meanwhile, *Oscillibacter* has been reported to be associated with trimethylamine oxide (TMAO), a risk factor for cardiovascular and cerebrovascular disease considered harmful by some researchers^36^. *Ruminococcus* has been considered positively and negatively related to health^37^, but most reports are negative. For example, inflammatory bowel disease (IBD) and metabolic disease patients showed increased levels of *Ruminococcus*^38,39^, suggesting a negative health impact. *Ruminococcus2* was reported to be positively correlated with body weight and serum lipid indices^40^. At the same time, *Ruminococcus2, Ruminococcus, Oscillibacter, Alistipes*, and *Parabacteroides* have been reported to be decreased in autoimmune disease patients^41^.

These results indicate that *Bifidobacterium bifidum*, a possible beneficial bacterium, increased when bathing in bicarbonate springs. However, there were also increases in bacteria with both positive and negative health effects when bathing in simple, bicarbonate and sulfur springs. In addition, the increased gut microbiota mostly differed by spa type.

In this study, the observed changes in *Bifidobacterium bifidum* after bathing in bicarbonate hot springs suggest potential health implications that need to be further investigated. This is consistent with recent findings regarding the potential mechanisms of the multifaceted effects of balneotherapy. Balneotherapy has been shown to affect systemic inflammatory mediators such as IL-1β, TNF-α, and IL-6, which are key players in inflammation and cartilage degradation in various rheumatic diseases^42,43^. Balneotherapy also appears to modulate stress biomarkers such as cortisol and eHsp72, contributing to its anti-inflammatory effects^42^. These findings and our results suggest a complex interplay of thermal and chemical factors in balneotherapy. The effects of hot spring bathing include physical effects due to heat and buoyancy, chemical effects due to components absorbed through the skin and mucous membranes, and spiritual effects due to changes in location and climate^44^. Among these, the thermal effect is the most significant^44^. For this reason, it is possible that the warming of the body and intestinal tract by hot spring bathing may increase the number and growth of favorable bacteria. However, the effects on the gut microbiota due to increased body temperature are still poorly understood^45^ and should be studied in the future. The absorbed substances from a hot spring may also have an effect on the changes in different bacterial species for each spa type in addition to the increase in body temperature. Simple springs may be less effective than other types of springs because they have a lower concentration of components.

One limitation of this study is that there is no non-intervention group. Future studies are needed to distinguish the effects of hot spring bathing by using a "no bathing control" or a "sauna control" in which body temperature is increased without hot spring bathing. Because this study design was a before-and-after comparison, further validation is needed through crossover studies or other means, including the addition of controls. In addition, although this study focused on changes in the gut microbiota, it is essential to evaluate the effects of hot springs on health indicators through future research to demonstrate their health benefits. It also remains to be determined to what extent the changes in the microbiome persist after bathing. In addition, no dietary or lifestyle interventions were directed in this study, and the participants were asked to live their everyday lives as usual. Therefore, the before-and-after comparison assumed that the gut microbiota is stable (robust) in healthy adults^46^. In addition, the reproducibility of the results needs to be studied in the future. In particular, further research should be conducted on sulfur and sulfate springs, where the number of participants was low in this study. Since the present study was conducted on Japanese subjects in the Kyushu region, generalizability to other racial groups was not ensured. Although the relative abundance of gut microbiota was used as an outcome in this study, future research on functional effects, such as the amount of short-chain fatty acids and other metabolites and immunoglobulin A (IgA), should help clarify the health benefits of hot spring bathing.

The results of this study suggest that bathing in bicarbonate springs may have health benefits by increasing the abundance of *Bifidobacterium bifidum*. In addition, an increase in several intestinal bacteria was found for each spa type, which may lead to different effects. This is the first study on the effects of hot spring bathing on the gut microbiota, and we believe it is highly novel. Further hot spring research could contribute not only to the improvement of public health but also to regional revitalization.

## Conclusion

An increased abundance of *Bifidobacterium bifidum* in healthy individuals was observed after seven consecutive days of bathing in bicarbonate springs. In addition, different bacteria were significantly increased after bathing in simple, bicarbonate, and sulfur springs. Further studies are expected to elucidate the contribution of hot spring bathing to health effects and mechanisms through the gut microbiota.

### Supplementary Information

Supplementary Information.

## Data Availability

The data that support the findings of this study are available from the author, S.M., upon reasonable request.
